# Factors predicting oral health behaviors among students age 13–15 years in Shushtar city, Iran

**DOI:** 10.1186/s12903-023-03363-7

**Published:** 2023-09-25

**Authors:** Seyedeh Zahra Marashi, Alireza Hidarnia, Seyedeh Somayeh Kazemi, Fatemeh Zarei

**Affiliations:** 1 Department of Health Education and Promotion, Faculty of Medical Sciences, Tarbiat Modares University, Tehran, Iran; 2 Department of Public Health, School of Health, Mazandaran University of Medical Sciences, Sari, Iran

**Keywords:** Oral health, Adolescents, Shushtar

## Abstract

**Background:**

Tooth decay and other oral health (periodontal) diseases are highly prevalent worldwide and present a significant economic burden. Oral health is particularly important for adolescents, as the World Health Organization has identified tooth decay as one of the most pressing global health issues. This study aims to identify predictors of oral health behaviors among students aged 13–15 years in the city of Shushtar, Iran.

**Methods:**

The present research is a cross-sectional study that was conducted during the summer of 2022 on 415 adolescents from the first secondary school in Shushtar. Two standard questionnaires were used to collect data, consisting of demographic information and questions related to measuring awareness, attitude, performance, and self-efficacy regarding oral health behaviors among adolescents. The collected data was analyzed using SPSS 22 statistical software through descriptive statistics, Pearson correlation, and regression analysis.

**Results:**

The average scores for awareness, attitude, self-efficacy in brushing teeth, self-efficacy in flossing, self-efficacy in going to the dentist, and adolescents’ performance on oral health behaviors are 5.72 ± 2.06, 36.40 ± 6.36, 25.40 ± 7.49, 14.15 ± 6.06, 15.80 ± 5.59, and 14.01 ± 4.02, respectively.

**Conclusion:**

Based on the results of this study, it can be concluded that adolescents have low levels of awareness, performance, self-efficacy, and a positive attitude towards oral health. Considering the significance of adolescence as a stage for shaping oral health behaviors and their impact on adulthood, it is recommended to increase awareness among adolescents and improve their health behaviors by conducting educational classes in schools.

## Background

Oral health is an essential component of overall health for every individual. It can impact one’s overall health by causing toothache, difficulty in eating, and changes in speech that affect the quality of life [1, 2]. Oral health care should consist of safe, consistent, diverse, accessible, cost-effective, and high-quality care that prevents or eliminates disease, pain, and infection. The most crucial index that indicates the level of people suffering from caries is related to the DMFT^1^ index. To calculate the DMFT of a community, the number of decayed, filled, and extracted teeth of the individuals in that community is counted, and the average is calculated [3–7].

Tooth decay and other oral health (periodontal) diseases have a high global prevalence and are considered a significant economic burden [8]. Unlike other infectious diseases, they cannot be stopped by taking antibiotics and can affect numerous teeth in a short duration. The World Health Organization (WHO) considers oral health as a prerequisite for maintaining public health throughout one’s life and emphasizes its importance more than ever [9, 10].

The period of transition from childhood to adulthood is associated with many changes, including physical, sexual, and psycho-social alterations, and unfortunately, neglecting healthcare, including oral health care, has become prevalent during this time [11, 12]. In this period, independence from parents increases, leading to changes in behavior, such as eating habits, smoking, health behaviors, lifestyle, which can all impact oral health and create lifelong habits [13].

Oral health is so vital to the adolescent age group that the WHO has declared tooth decay as one of the most critical health problems in the world, with a prevalence of 60–90% among school students. Additionally, oral health diseases affect nearly 3.5 billion people globally [12, 14, 15]. A study conducted in collaboration with the WHO and the Ministry of Health showed that the DMFT index in Iran is 67.1%, with girls having a higher index than boys, and half of the students refuse to brush their teeth. Studies in Ahvaz demonstrated that tooth decay is more prevalent among girls, and most students, particularly boys, are at high risk of tooth decay [16–18].

In addition to non-behavioral factors such as the shape and form of teeth, placement of teeth, type of teeth, etc., and genetics, hygiene behaviors in the field of oral health, including brushing at least twice a day, using dental floss, using mouthwash, and undergoing dental examinations, are essential for improving oral health, preventing and controlling tooth decay and other oral health (periodontal) diseases [19–21].

It should be noted that the risk of oral health diseases increases when awareness and oral health measures are ignored. Improper nutritional behavior in students, such as high sugar consumption, not consuming dairy products, and internalizing habits such as reluctance to brush teeth and not using dental floss, can lead to a high incidence of tooth decay [22].

Given the significance of oral health and lifestyle habits in enhancing oral health status, the susceptibility of teenagers to promote health during this pivotal period, the limited indicators of oral health behaviors, and the inadequate examination and care for students’ oral health in Shushtar city, Iran, this study aims to identify the predictors of oral health behaviors among 13-15-year-old students in the aforementioned city.

## Methods

The present research is a cross-sectional descriptive-analytical study that was conducted in 1401 on 415 girls aged 13–15 years studying in Shushtar. The study adhered to the Declaration of Helsinki and received ethical approval from the Human Ethics Committee at the University of Tarbiat Modares, Tehran, Iran (IR.MODARES.REC.1401.125). Written informed consent was obtained from all parents and/or legal guardians for their children’s participation in the study.

Shushtar has 27 urban schools with a total enrollment of 4,881 students in grades one to secondary school. The sampling method used for this research was multi-stage random sampling based on Cochran’s formula, which required a sample size of 415 individuals. Therefore, six urban girls’ schools were selected from the aforementioned schools, and a specific number of students were randomly chosen from each school based on the student population and the required total sample size.

The inclusion criteria for the study were students aged 13–15 years old, residents of Shushtar City, absence of oral diseases and underlying diseases, currently enrolled in school, and informed consent from both students and their parents. Exclusion criteria included not meeting at least one of the aforementioned criteria. In research involving minors (under 18 years of age), the issue of informed consent primarily revolves around parental rights. Accordingly, the parents provided their consent for their children’s participation in the study via a phone call, followed by signing the consent forms during a scheduled visit to the school. The “consent students” meant positive consent to participate in the study, which was done verbally.

The data collection instruments comprise of demographic characteristics and two questionnaires. The first questionnaire, consisting of 36 questions, evaluates the awareness, attitude, and behavior towards oral health among adolescents. The demographic characteristics include age, education level of parents, number of family members, economic status of the family, and frequency of brushing and flossing daily.

The awareness section of the questionnaire contains 12 questions, with a score of one given for the correct option and zero for other options. The range of scores is between 0 and 12, with a higher score indicating greater awareness. The attitude section has 10 items and uses a 5-option Likert scale ranging from completely agree (score 5) to completely disagree (score 1). The range of scores is between 10 and 50, with a higher score indicating a better attitude. The performance section has 14 items measured as yes, sometimes, and no options. Two items with negative correlation were removed, resulting in a score range of 0–24. Higher scores indicate better performance regarding oral health. The validity and reliability of the questionnaire were confirmed by Yavari et al. [23], and the reliability was reconfirmed in this study (α > 0.7).

Oral Health Self-Efficacy Questionnaire [24]: This questionnaire consists of three parts assessing the respondent’s confidence in their ability to brush their teeth (10 questions), floss (7 questions), and visit a dentist (7 questions). The questionnaire is based on the theoretical definition of self-efficacy, which refers to an individual’s ability to understand, learn, or perform actions at specified levels.

In this questionnaire, participants were asked to rate their confidence level using a four-point Likert scale (4 = completely sure, 3 = somewhat sure, 2 = not quite sure, and 1 = not completely sure) in different situations related to brushing, flossing, and visiting the dentist. The scores for self-efficacy in brushing teeth ranged from 10 to 40, while scores for self-efficacy in flossing and visiting the dentist ranged from 7 to 28 each.

The reliability and validity of the questionnaire have been confirmed in previous study [25], and in Iran, its reliability was reported in Ardakani et al.‘s study [26] with a Cronbach’s alpha coefficient of 0.79. The present study also confirmed the reliability of the questionnaire (α > 0.7).

Prior to completing the questionnaire, the researcher explained the study objectives, assured confidentiality of information, obtained written informed consent from all parents and/or legal guardians of participants, and allowed them to withdraw from the study at any stage. Data collected using SPSS software version 22 underwent normality testing using the Kolmogorov-Smirnov test before being analyzed using descriptive statistics, Pearson’s correlation test, and regression analysis to predict the dependent variable at a significance level of ≤ 0.05.

## Results

In the present study, 415 adolescents aged 13 to 15 years with an average age of 13.69 ± 0.75 participated (see Table 1). The average scores for awareness, attitude, performance, self-efficacy in brushing teeth, self-efficacy in flossing, and self-efficacy in going to the dentist were 5.72 ± 2.06, 36.40 ± 6.36, 14.01 ± 4.02, 25.40 ± 7.49, 14.15 ± 6.06, and 15.80 ± 5.59, respectively (see Table 2).

**Table 1:** Frequency and relative frequency of demographic variables

		Number	percent
Father’s education	Less than a diploma	160	38.6
	Diploma and more	222	53.5
Mother’s education	Less than a diploma	140	33.7
	Diploma and more	240	57.8
Economic situation	very good	94	22.7
	weak	13	3.1
Number of brushing teeth	Once and less	217	52/3
	Twice and more	192	46.3
Number of flossing	Once and less	353	85
	Twice and more	52	12.5
Condition of the teeth	Healthy	266	64.1
	Decay/Missing/Filling	135	32.6
Family members	Less than 4 people	34	8.2
	4 people and more	343	82.6
Average age of participated = 13.69 ± 0.75			

**Table 2:** Mean, standard deviation, and maximum obtainable score in oral and dental health behaviors of adolescents

The dependent variables	Mean &SD	Percentage of score obtained	The maximum score that can be obtained
Awareness	5.72 ± 2.06	47.6	12
Attitude	36.40 ± 6.36	72.8	50
Performance	14.01 ± 4.02	58.3	24
Self-efficacy in brushing	25.40 ± 7.49	63.5	40
Self-efficacy in flossing	14.15 ± 6.06	50.5	28
Self-efficacy in going to the dentist	15.80 ± 5.59	56/4	28

The findings related to the awareness of adolescents about oral health showed that 90% of adolescents considered brushing to be very important, while 32% considered brushing after meals to be important. In addition, 26% considered flossing before brushing to be important, and 23% believed using mouthwash after brushing was important.

According to 44% of adolescents, brushing is effective in preventing tooth decay, while only 7% of them considered brushing to be effective in preventing heart disease.

In general, 28% of adolescents admitted to brushing their teeth, while only 39% brushed their teeth before going to bed. Among those who reported regularly changing their toothbrushes and going to the dentist for examination, the percentages were 28% and 39%, respectively.

Regarding self-efficacy, the findings revealed that 34% of adolescents were confident in their ability to brush their teeth when tired, 39% when they had a headache, and 35% when they were sick. As for the use of dental floss, 48% of adolescents used it when they were tired, 38% when they did not intend to visit the dentist, 35% when on vacation, 44% when they had a lot of work, 48% when they had a headache, and 49% when they had complete confidence in using dental floss during illness. Finally, 38%, 37%, and 32% of adolescents were fully confident in going to the dentist even under bad economic conditions, when they were busy, and when they had an uncomfortable dental experience, respectively (see Table 3).

**Table 3:** Adolescents’ Awareness, Attitude, Performance, Self-efficacy in brushing, Self-efficacy in flossing and Self-efficacy in going to the dentist towards oral health behaviors

variables	question	percent
**Awareness**	The importance of brushing	90%
	Brushing time after meals	32%
	Floss before brushing	26%
	Use mouthwash after brushing	23%
**Attitude**	The effect of brushing in the prevention of tooth decay	44%
	The effect of brushing in the prevention of heart disease	7%
**Performance**	teeth brushing	28%
	Brushing before going to bed	39%
	Changing the toothbrush	28%
	See a dentist for an examination	39%
**Self-efficacy in brushing**	Tired time	34%
	headache time	39%
	time of illness	35%
**Self-efficacy in flossing**	Tired time	48%
	When they do not intend to go to the dentist	38%
	vacation time	35%
	having a lot of work	44%
	presence of headache	48%
	time of illness	49%
**Self-efficacy in going to the dentist**	bad economic situation	38%
	having a lot of work	37%
	Having a bitter dental experience	32%

Pearson’s correlation analysis indicated a significant and positive correlation between awareness, attitude, performance, self-efficacy in brushing, self-efficacy in flossing, and self-efficacy in going to the dentist (p < 0.05) (see Table 4).

**Table 4:** Pearson’s correlation coefficient and significance level between the mean scores of the dependent variables

age			awareness	Attitude	Performance	Self-efficacy in brushing	Self-efficacy in flossing	Self-efficacy in going to the dentist
age	r	1	0.103*	0.129**	0.056	0.088	0.043	0.050
	p	1	0.036	0.008	0.254	0.074	0.383	0.312
awareness	r	0.103	1	0.426**	0.249**	0.299**	0.179**	0.151**
	p	0.036	1	0	0	0	0	0.002
Attitude	r	0.129**	0.426**	1	0.277**	0.304**	0.184**	0.163**
	p	0.008	0	1	0	0	0	0.001
Performance	r	0.056	0.249**	0.277**	1	0.532**	0.458**	0.336**
	p	0.254	0	0	1	0	0	0
Self-efficacy in brushing	r	0.088	0.299**	0.304**	0.532**	1	0.545**	0.532**
	p	0.074	0	0	0	1	0	0
Self-efficacy in flossing	r	0.043	0.179**	0.184**	0.458**	0.545**	1	0.545**
	p	0.383	0	0	0	0	1	0
Self-efficacy in going to the dentist	r	0.050	0.151**	0.163**	0.336**	0.532**	0.545**	1
	p	0.312	0.002	0.001	0	0	0	1

To better understand the factors influencing oral health behaviors among 13-15-year-old adolescents, a multivariate regression analysis was performed. The constructs of awareness, self-efficacy in brushing teeth, and self-efficacy in using dental floss collectively predicted 24.5% of the dependent variable (see Tables 5 and 6).

**Table 5:** Summary of the multivariate regression model of oral health behaviors in adolescents

R Square	Adjusted R Square	F	Sig
0.254	0.245	27.856	0.0001

**Table 6:** Regression analysis of factors related to oral health behavior in adolescents

Variables	β	P value	The dependent variable
Awareness	0.104	0.255	Oral health performance
Attitude	0.045	0.129	
Self-efficacy in brushing	0.159	0.000	
Self-efficacy in flossing	0.153	0.000	
Self-efficacy in going to the dentist	-0.024	0.517	

## Discussion

The aim of this study was to examine the predictors of oral health behaviors among 13–15 year old students in Shushtar city, Iran. Based on the research findings, adolescents lacked sufficient awareness in the field of oral health, with only 47% exhibiting awareness of oral health practices. While over 90% of adolescents recognized tooth brushing as essential for maintaining good oral health, only 26% and 23% were aware of the importance of dental flossing and mouthwash, respectively. Mishra et al.‘s [27] study on parental awareness and children’s dental status found that low parental awareness can negatively impact oral health in children. Similarly, Abdulbaqi et al. [28] and Al-Qahtani et al. [29] demonstrated that people generally have inadequate awareness of oral health, and recommended government educational programs and health awareness campaigns in elementary and high schools. Tadin et al.‘s [30] research indicated that students whose families had a dentistry background exhibited better awareness of oral care than other students. Regarding differences in results between this study and others, factors such as age group composition, cultural disparities between developed and underdeveloped countries, and varying levels of family awareness in Shushtar city may account for such variations.

It is crucial to assess the level of awareness among both parents and students when it comes to oral health. Parents can serve as guides for their children in promoting healthy behaviors, a factor that was not considered in this study. Attitude is considered a significant factor in initiating and maintaining good health behaviors [31]. In this study, adolescents demonstrated a positive attitude toward oral health behaviors, with over 72% exhibiting favorable attitudes. Similar studies conducted by Deng et al. [32], He et al. [33], and Yilmaz et al. [34] also reported positive attitudes among students despite insufficient awareness and practices related to oral health. However, Lawal et al. [35] found poor attitudes among students regarding oral health, which may be attributed to living in low-income areas with limited access to education and healthcare facilities.

Despite a lack of adequate awareness regarding oral hygiene practices, individuals generally hold a positive attitude toward this matter. This can be attributed to the desire among people, especially adolescents who are at a sensitive age and highly value physical appearance, to maintain healthy and aesthetically pleasing teeth. Nonetheless, it appears that this positive attitude does not always translate into appropriate behavior. According to researchers, self-efficacy is defined as the result of enduring challenges and trusting in one’s abilities [36]. In the present study, adolescents demonstrated poor self-efficacy in brushing teeth, flossing, and going to the dentist, with self-efficacy exhibited in only about 50% of behaviors. The results of the study by Dolatabadi et al. [37] showed high self-efficacy in brushing teeth among teenage girls. Additionally, Mizutani et al. [38] found that high self-efficacy among adolescents had a positive impact on oral health behaviors, while Parker et al. [39] and Allen et al. [40] noted low self-efficacy among their target groups and acknowledged that those with low self-efficacy were more likely to experience gum bleeding problems. Therefore, self-efficacy, as a strong predictor, can encourage people to perform health behaviors. These differences seem to be related to the importance given to oral health in different countries and regions. If adolescents receive sufficient information in schools and become aware of the importance of brushing, flossing, and going to the dentist, they can develop high levels of self-efficacy through frequent practice. Also, some studies have compared the oral health of male and female adolescents, while the target group in the present study consisted only of female adolescents. Furthermore, the current study focused specifically on self-efficacy in brushing teeth, flossing, and going to the dentist among adolescents, while other studies may have examined different dimensions of self-efficacy.

In the present study, the final goal of oral health performance was not adequately met, with only 58% of oral health behaviors being performed. However, Gharlipour et al. [41] demonstrated that appropriate interventions can improve the performance of adolescents in terms of oral health, even if they are not initially at an optimal level. Similarly, Tudoroniu et al. [42] and Abu-Gharbieh et al. [43] found that people are generally not satisfied with good performance in the field of oral health, which is consistent with the results of the present study. Costa Pazos et al. [44] discussed the importance of self-esteem in adolescents and noted that those with high self-esteem tend to perform oral health behaviors well.

## Conclusion

Based on these findings, it can be deduced that achieving optimal oral health outcomes, which are closely linked to individuals’ behavior, is influenced by various factors and dimensions. The effectiveness of interventions and the environmental context in which people live also play crucial roles in shaping oral and dental health. In regions where health policies and investments in health promotion are not prioritized and where matters concerning oral and dental health are inadequately communicated to the public, particularly adolescents, suboptimal outcomes and reduced expectations may result. Such circumstances not only compromise adolescents’ future oral health but also have negative effects on their overall well-being.

Therefore, based on the results of this research, it is recommended to increase awareness among adolescents and improve health behaviors through educational classes held in schools. This is especially important given the significance of the adolescent age group and its impact on the formation of oral and dental health behaviors in adulthood. Addressing the issues of lack of awareness, poor performance, low self-efficacy, and inadequate attitudes towards oral health among adolescents requires a comprehensive approach that involves education, accessible healthcare facilities, and effective policy implementation.

### Limitations of the study

There are several limitations to this study, including self-reporting by students when completing the questionnaires, internet connectivity issues, and non-cooperation from some school principals. To address these limitations, necessary arrangements were made in coordination with the Department of Education in Khuzestan and Shushtar.

## Data Availability

The data will be available from the corresponding author on request.

## Footnotes

1 Decayed, Missing, and Filled Teeth (DMFT).
